# Anterior Thigh Tissue Thickness Measured Using Ultrasound Imaging in Older Recreational Female Golfers and Sedentary Controls

**DOI:** 10.3390/geriatrics2010010

**Published:** 2017-02-07

**Authors:** Isabel Herrick, Simon Brown, Sandra Agyapong-Badu, Martin Warner, Sean Ewings, Dinesh Samuel, Maria Stokes

**Affiliations:** 1 Faculty of Health Sciences, Building 45, University of Southampton, Southampton SO17 1BJ, UK; musclestudy2014@gmail.com (I.H.); simon.brown@soton.ac.uk (S.B.); s.agyapong-badu@bham.ac.uk (S.A.-B.); m.warner@soton.ac.uk (M.W.); sean.ewings@soton.ac.uk (S.E.); d.samuel@soton.ac.uk (D.S.); 2 Arthritis Research UK Centre for Sport, Exercise and Osteoarthritis, Nottingham NG7 2UH, UK; 3 School of Sport, Exercise and Rehabilitation Sciences, University of Birmingham, Edgbaston, Birmingham B15 2TT, UK; 4 Southampton Statistical Sciences Research Institute, University of Southampton, Southampton SO17 1BJ, UK

**Keywords:** thigh tissue thickness, quadriceps muscle, ultrasound imaging, ageing, golf, physical activity

## Abstract

Physical activity is vital for the prevention of sarcopenia and frailty. The training effects of recreational golf on muscle function in older people are unknown. The present study examined quadriceps muscle and subcutaneous fat thickness in 66 older females. Thirty-one golfers (mean age 69.1 years, standard deviation ±3.4) were compared with 35 less active non-golfers (73.4 ± 4.2 years). Images of the dominant anterior thigh were obtained using real-time B-mode ultrasound imaging. Thickness of muscle (rectus femoris, vastus intermedius, and intermuscular fascia) and subcutaneous tissue (fat and perimuscular fascia) was measured, and percentage contributions calculated. Muscle thickness was significantly greater (*p* < 0.001) in golfers (mean 2.78 cm ± 0.73 cm) than non-golfers (2.18 cm ± 0.55 cm). Mean percentage contribution of muscle and non-contractile tissue was 64% ± 9% and 36% ± 9%, respectively, in golfers, compared to 58% ± 8% and 42% ± 8% in non-golfers (*p* = 0.013). Multiple linear regression analysis, controlling for age and BMI, showed that golfers still had higher total anterior thigh thickness (regression parameter for non-golfers B = −0.984, *p* = 0.004) and higher muscle thickness (B = −0.619, *p* = 0.002). This study indicates an association between recreational golf and greater relative thigh muscle thickness and lower subcutaneous fat than in less active controls. Training effects need to be examined in prospective controlled trials in males and females in different age groups.

## 1. Introduction

Loss of muscle mass and strength, and the resulting loss of function that occur in sarcopenia begins at about the age of 40 years, and accelerates beyond 65–70 years [1]. Rates of decline can be slowed with regular strenuous training in masters athletes [2,3]. However, little is known about the effects of recreational activity on muscle in older people in the general population.

Quadriceps is an important muscle for mobility, and its relative thickness to subcutaneous fat has been quantified in healthy young and older adults who are not very active [4]. Studying thigh tissue thickness in groups with different activity levels may provide an indication of musculoskeletal health using the simple, rapid measurement of relative muscle thickness on ultrasound scans.

Recreational golf is associated with better cardiovascular health and musculoskeletal function [5] and balance [6] than sedentary controls, and greater life expectancy [7]. The specific effects of playing golf on aspects of musculoskeletal health warrant investigation.

Females have greater loss of muscle thickness (12%–23%) and strength (58%–68%) than males [8]. Agyapong-Badu et al. [4] reported that older females (aged 65–90) have a mean anterior thigh percentage muscle thickness of almost 20% (18%) less than males in the same age category. Sarcopenia can be partially reduced by physical activity; resistance exercise being the most effective type of training [2].

Ultrasound is suitable for routine imaging, as it is safe (contains no ionising radiation), rapid, portable, and cost-effective [9]. B-mode ultrasound imaging (USI) is a reliable (within and between investigators) method of measuring various skeletal muscles and sub-cutaneous tissue, when used by an experienced operator following strict protocol guidelines [9,10,11]. Ultrasound imaging of quadriceps has been shown to be valid when compared to magnetic resonance imaging (MRI) [9,12,13,14]. Muscle mass (cross-sectional area, CSA or thickness) and strength are highly correlated [8,15]. Therefore, measuring the thickness of a muscle will provide an estimate of its strength.

The World Health Organisation recommends that those over 65 years should carry out muscle strengthening activities on two or more days per week [16]. It is unknown whether playing golf would have sufficient strength training effects, and this knowledge gap has been highlighted as an urgent research need, particularly in older people [17].

The present study aimed to use B-mode USI to compare the relative proportions of quadriceps muscle and non-contractile (subcutaneous fat, SF, and perimuscular fascia) tissues to total anterior thigh thickness between moderately active and less active older females. The study addressed the question: is moderate activity in recreational golfers associated with greater relative muscle thickness and less subcutaneous fat than in less active older females? A positive outcome would warrant a prospective study to examine the strengthening effects of playing golf, as muscle size is an indicator of strength [8,15].

## 2. Materials and Methods

This was a cross-sectional, quantitative, observational comparative study.

### 2.1. Participants

A convenience sample of 31 female golfers (aged 65–77 years, mean 69.1 ± 3.4) were recruited from golf clubs in Hampshire. Inclusion criteria were: female golfer aged 65–80 years who played a minimum of one round of golf (18 holes) per week, walking between holes. Three questions from the Physical Activity Scale for the Elderly (PASE) were used for screening purposes to ensure that participants were moderately active. Questions included: “In a typical week, how many days would you participate in light sport/recreational activities (e.g., light cycling, lawn bowls, golf with buggy)?; In a typical week, how many days would you participate in moderate sport/recreational activities (e.g., doubles tennis, golf without a buggy, ballroom dancing)?; In a typical week, how many days would you participate in strenuous sport/recreational activities (e.g., singles tennis, swimming, jogging)?” An additional question for the golfers was: “Do you play a full round of golf (18 holes), by walking, every week? If so, how many times per week?” Participants were not requested to specify their activities other than golf, as the purpose was to ensure that they were moderately active and not considered sedentary, for inclusion in the study.

Participants were cognitively able and capable of travelling to the testing location. Those with common conditions (e.g., diabetes/blood pressure) were included providing that their condition was controlled and had not deteriorated recently. Exclusion criteria were: musculoskeletal injury in the last five years which led to immobility for more than one week; uncontrolled diabetes or blood pressure; a known neurological disorder; arthritis which restricted the ability to perform every-day activities; receiving treatment for cancer; taking medication which affects muscle function. These criteria aimed to include those whose thigh tissue thickness would represent a moderately active older population, unaffected by pathology. Ethical approval was gained from the Faculty of Health Sciences, University of Southampton Ethics Committee (Ethics approval number 9650). Participants provided written, informed consent prior to data collection.

The present participants were compared with a subset of non-golfing older females from a larger study of sedentary young and older males and females [4] whose scans were re-analysed using a modified measurement protocol (see below). The above inclusion (except regular moderate activity) and exclusion criteria applied to this group. The characteristics of both groups of participants are shown in Table 1.

### 2.2. Ultrasound Imaging

**Figure 1:**
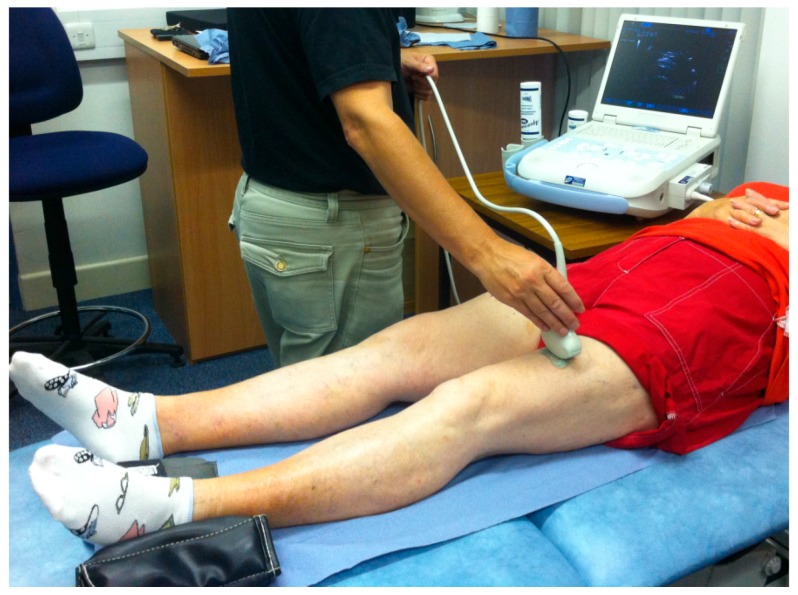
Ultrasound imaging set up with the participant in supine.

Participants rested while seated for a minimum of 10 minutes before the ultrasound images were taken. Images of the anterior thigh were produced using a real-time B-mode ultrasound scanner (Imagic Agile, Pie Data, Ltd., Kontron Medical, St Germaine en Laye, France) with a 5–6.6 MHz curvilinear (abdominal) transducer. The transducer was placed transversely at a point two-thirds of the distance between the anterior-superior iliac spine and the superior pole of the patella, which was marked on the skin with a non-toxic pen [18]. Participants lay relaxed in supine lying, with the knee extended and leg supported by sand bags on either side of the ankle to maintain the hip in neutral (Figure 1). The probe was placed on the skin, with minimal pressure, to produce an image showing rectus femoris (RF), vastus intermedius (VI), SF, and perimuscular fascia. Two images were taken from the dominant side by the same experienced operator (SB). Leg dominance was determined by the preferred leg for kicking a ball.

**Figure 2:**
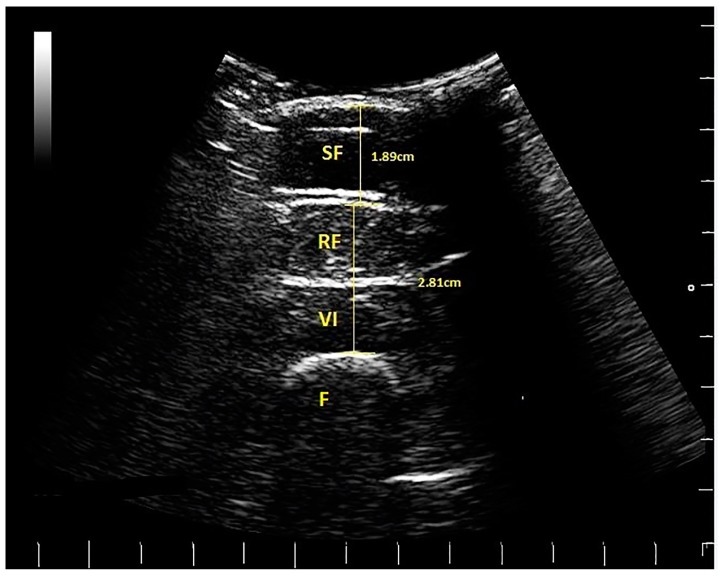
Ultrasound scan from a female golfer, indicating subcutaneous fat (SF), rectus femoris (RF), vastus intermedius (VI) and the femur (F). The scale on the right and bottom of the scan is in cm (white lines). The thickness of the tissues is indicated (SF = 1.89 cm) and muscle (RF plus VI = 2.81 cm).

Images were measured later off-line by IH, using a Matlab algorithm (written by MW). The thickness of two tissue layers was measured: (1) the superficial non-contractile layer comprised subcutaneous fat and superficial fascia measured from the skin to the inferior border of the superficial fascial layer; (2) the contractile layer consisted of RF and VI muscles, including the deep fascia between them, measured from the inside edge of the superior border of RF to the inside edge of the inferior border of VI (Figure 2). These two measurements were a simplified version of the measurement protocol used by [4], which involved making separate measurements of subcutaneous fat, superficial, and deep fascia and muscle thickness; hence, scans from the earlier study were re-analysed using the modified protocol. Each image was measured twice, and the mean values were used in the analysis.

### 2.3. Statistical Analysis

Data collected on the dominant leg in the current study were compared with re-analysed scans from a subset of participants studied by [4], who were predominantly sedentary females aged 66 to 80 years. In the previous study, SAB—an experienced USI operator—had established the intra-rater between-day reliability of the USI technique (i.e., obtaining images and making measurements) [4].

During training prior to the present study, IH examined intra-rater reliability of measuring the same scans (*n* = 20) on two different days, and examined inter-rater reliability against the experienced investigator (SAB) who measured the same 20 scans. Intra-rater reliability of quadriceps muscle and SF thickness measured by IH was excellent, with an intraclass correlation coefficient (ICC 1,1) of 0.99 (95% confidence intervals (CI) of 0.987 to 0.996) for muscle and 0.99 (95% CI (0.990 to 0.997) for SF. Inter-rater reliability was also excellent: ICC (2,2) of 0.99 (95% CI (0.947 to 0.997) for muscle and 0.99 (95% CI (0.988 to 0.998) for SF.

Data were imported from Microsoft Excel and analysed using SPSS 21 (SPSS Inc., Chicago, IL, USA). Baseline characteristics of the golfers and non-golfers were analysed. Histograms revealed a non-Gaussian distribution for age and BMI for golfers and non-golfers. Therefore, the non-parametric Mann–Whitney U test was used to compare participant characteristics between groups.

Mean values for each soft tissue layer from the two images taken were used in the analysis. Percentage proportions of superficial non-contractile and deep muscle layers to total anterior thigh thickness were calculated. Distribution of ultrasound data was found to be normal using histograms. Independent samples *t*-tests were used to compare data between the golfers and sedentary older females. Multiple linear regression analyses compared thigh tissue thickness between golfers and non-golfers, whilst controlling for age and BMI.

## 3. Results

### 3.1. Participant Characteristics

Mann–Whitney U analysis revealed that the golfers were younger (median = 68 years) than the non-golfers (median = 74), and had lower BMI (golfers median = 24.8 kg/m^2^; non-golfers median = 27.7), and that these differences were significant (*p* < 0.0005). The weight of golfers (median = 64) was significantly less (*p* = 0.002) than that in non-golfers (median = 72.7).

The distribution of age and BMI in the two groups is displayed in Table 2, which shows more golfers in the lower age groups and BMI categories than non-golfers.

All golfers participated in moderate activity at least once a week (*n* = 31), compared to only 28% (*n* = 9 of the 32) of non-golfers. Three non-golfers did not complete the PASE. Golfers were moderately active for a median of 3.5 days per week (some doing activities in addition to golf), which was significantly greater (*p* < 0.0005) than non-golfers (median = 0 days per week), and was intended to compare groups with differing levels of habitual physical activity.

### 3.2. Ultrasound Parameter Measurements

Independent *t*-tests revealed that golfers had significantly greater total anterior thigh thickness, higher actual and percentage muscle thickness, higher muscle thickness per kg of body mass, and lower percentage subcutaneous non-contractile tissue compared to non-golfers (Table 3).

**Figure 3:**
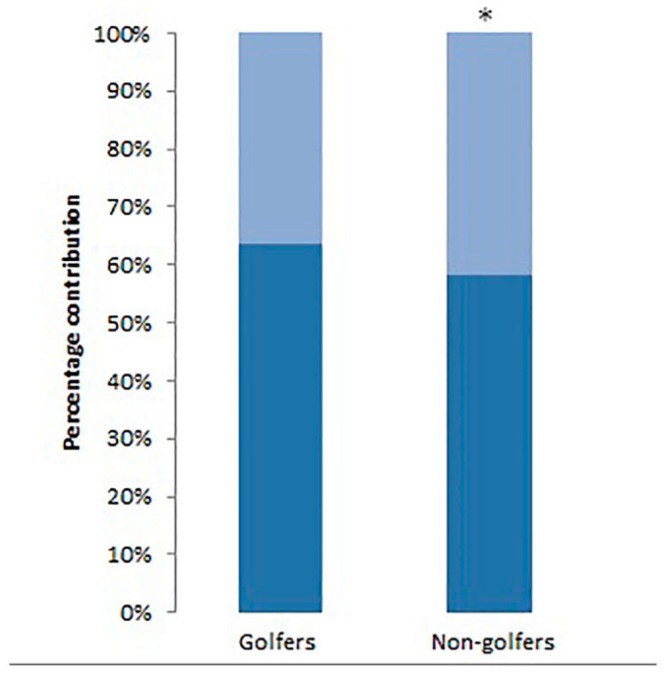
Percentage contribution of muscle thickness plus deep fascia (dark blue) and subcutaneous fat plus superficial fascia (light blue) in golfers and non-golfers. * Difference between groups was statistically significant (*p* < 0.05).

Difference in percentage contributions between golfers and non-golfers is shown in Figure 3.

Since there were significant differences in characteristics between golfers and non-golfers, multiple linear regression analysis examined the influence of age and BMI on the ultrasound findings. Total anterior thickness was higher in golfers (B = −0.984, *p* = 0.004), controlling for BMI and age (Table 4). Muscle thickness was also higher in golfers (B = −0.619, *p* =0.002), so age and BMI did not influence these soft tissue measurements on ultrasound scans.

## 4. Discussion

The relative contributions of quadriceps muscles and non-contractile tissue (subcutaneous fat and perimuscular fascia) to total anterior thigh thickness were quantified using USI in a group of moderately active older female golfers and were compared with non-golfers. Simple rapid ultrasound measurements showed that the golfers had greater proportions of muscle thickness and leaner thighs than the non-golfers, indicating an association with the moderate activity of recreational golf; however, controlled studies are needed to determine if there is a training effect on the thigh muscles from playing golf. These findings suggest that golf may be sufficient to enable older females to achieve the strengthening effects of moderate activity recommended by the World Health Organisation [16], but the possibility of other factors contributing to the muscle size differences between the groups needs to be studied.

Relative thigh tissue thickness of muscle and SF, measured using ultrasound imaging, may be a useful indicator of musculoskeletal health status. Golfers had a higher percentage of muscle thickness and actual muscle thickness, and a lower percentage of subcutaneous fat and fascia compared to non-golfers (Table 3). This may indicate that moderate activity in older females is associated with leaner thighs (less SF in relation to muscle thickness), which has implications for health and physical function. Muscle thickness (cm) per kg of body mass was higher (*p* < 0.0005) in golfers (0.44) than non-golfers (0.30). This indicates that in the groups studied, when a golfer and non-golfer of the same weight were compared, the golfer was likely to have (0.14 cm) more muscle per kg in the anterior thigh. The greater muscle thickness in golfers was expected, as golf is known to improve musculoskeletal function [5], but the present findings were the first to provide quantitative evidence of greater muscle size associated with playing golf.

The fact that there were differences in age and BMI between the groups did not explain the differences in tissue thickness measurements. Multiple regression analyses that controlled for the difference in age and BMI between the golfers and non-golfers showed that total anterior thigh thickness was almost 1 cm thicker in golfers compared to non-golfers, and muscle thickness was 0.6 cm thicker in golfers compared to non-golfers. As 28% of the non-golfers participated in moderate activity at least once a week and all volunteers were self-selected, even those who did not participate in moderate activity may be more active than those who would not volunteer for such a study. The difference in relative thigh tissue thickness between active and sedentary females may therefore be even larger when compared to a truly sedentary population.

The reliability and validity of ultrasound measures of thigh tissues have been established. The intra- and inter-rater reliability of muscle and non-contractile tissue thickness measurements made by IH and SAB on the same scans in a preparatory study were excellent (>0.99). Therefore, the thickness measurements in golfers (measured by IH) and non-golfers (measured by SAB) can be compared confidently. Evidence indicates that USI is valid when compared to MRI in the anterior thigh [13], rectus femoris [9], vastus medialis [14], whole quadriceps volume [12], and when measuring subcutaneous fat [11].

There are potential uses of measuring thigh tissue thickness with ultrasound imaging in a number of environments, as the technique is rapid, safe, and cost-effective [9]. Ultrasound is feasible for use in a clinical setting; e.g., to monitor thigh tissue thickness in the aging population to try to identify those losing muscle mass rapidly, who are at risk of functional decline and loss of independence. When monitoring weight loss, USI could ensure fat and not muscle was being lost and conversely during weight gain, that muscle was increasing and not subcutaneous fat. This scenario is also relevant to patients in intensive care to monitor muscle wasting and recovery, as demonstrated by [19].

The previous study of sedentary groups by [4] measured muscle thickness by excluding the inter-muscular fascia between RF and VI, but for ease and efficiency of measuring images in a clinical or field setting, the protocol used in the present study is advised for clinical use. The modified protocol includes the superficial fascia in the subcutaneous fat measurement, and includes the deep fascia in the muscle thickness measurement. This simple method of measuring means that images are easier and quicker to measure, as well as avoiding fascial measurements, which are less reliable than muscle measurements [4].

A limitation of including fascial measurements is that the amount of muscle and subcutaneous tissue will be over-estimated. However, this limitation is outweighed by the benefits of the simpler measuring method, as it may allow clinicians to use ultrasound imaging more regularly. Another limitation of using USI is that it is not possible to measure and subtract intramuscular fat from muscle tissue, so muscle thickness will be overestimated. MRI is considered to be the gold standard for identifying intramuscular fat, and provides a more accurate measure of total fat content [20], but is not feasible in field environments.

The thigh was only measured at one site, so this may not represent the whole thigh. One site was measured in the current study, as this is the protocol that clinicians are likely to follow, due to time constraints. Further research into the number of sites required to reflect whole body composition is warranted.

The present cross-sectional study comparing golfers—who were self-selected—with sedentary controls cannot be used to determine the training effects of golf, which needs to be examined in a randomised controlled prospective trial. Future studies should document levels and different types of physical activity undertaken by participants formally (which was a limitation of the present study), so that the true contribution of golf on training effects can be determined.

Golfers in the present study were compared retrospectively with sedentary controls from a prior related study; therefore, matching controls with golfers was not possible. This resulted in the non-golfers being an average of four years older than the golfers, although the multiple regression analysis did not demonstrate an effect of age on the difference in thigh tissue thickness. In the golfing group, the other moderate activities that some participants undertook could have contributed to the differences in muscle size found. There were also nine participants in the sedentary group who participated in moderate activity at least once a week, which did not represent a truly sedentary population. Other potential differences between the groups that were not examined in the present study include diet, socio-economic status, etc. Such confounding variables will need to be considered in future studies.

Further research is warranted to study relative thigh tissue thickness of other populations, such as females over 80, older male golfers, and active young people of both genders. A comprehensive database could then be used as a guide in clinical practice. It would also be interesting to compare relative thigh tissue thickness of participants in different sports. It would be useful to establish the relationship between tissue thickness of the anterior thigh—assessed at one site—with the rest of the body to determine whether the thigh reflects relative tissue thickness of the whole body. Regarding the training effect of recreational golf on thigh muscle size (an indirect measure of muscle strength), longitudinal studies are needed.

## 5. Conclusions

Relative thickness of quadriceps muscle was higher and subcutaneous non-contractile tissue thickness lower in moderately active older golfers, compared to less active older females. These findings provide the first objective indication that recreational golf is associated with larger quadriceps muscles (an indicator of strength), so a prospective study is warranted to see if there is a specific strengthening effect of golf, separate from other activities. Relative tissue thickness in the anterior thigh can be assessed simply using ultrasound imaging, which could be useful in clinical and field environments to assess those at risk of frailty and the effects of interventions.

## Figures and Tables

**Table 1:** Descriptive characteristics of golfers and non-golfers (from Agyapong-Badu et al. 2014 [4]).

Characteristic	Golfers (*n* = 31)	Non-Golfers (*n* = 35)
Age (years) *	69.2 ± 3.5 (65–77)	73.4 ± 4.2 (66–80)
Height (m)	1.6 ± 0.05 (1.5–1.7)	1.6 ± 0.06 (1.5–1.8)
Body Mass (kg) *	63.9 ± 5.4 (50–72)	71.6 ± 10 (53–94)
BMI (kg·m^−2^) *	24.8 ± 2.5 (19–30)	27.9 ± 3.5 (22–34)

Abbreviations: BMI body mass index. Values are Mean ± SD (range); * Significant differences between groups for age and BMI (*p* < 0.0005) and body mass (*p* = 0.002).

**Table 2:** Age group and BMI categories of golfers and non-golfers.

**Age Group**	**Golfer**	**Non-Golfer**
65–69	20	9
70–74	7	11
75–80	4	15
**BMI Category**	**Golfer**	**Non-Golfer**
Underweight	1	0
Healthy	16	9
Overweight	14	16
Obese	0	10

**Table 3:** Ultrasound measurements of non-contractile and contractile layers of the dominant anterior thigh in golfers and non-golfers.

	Golfers Dominant Thigh *n* = 31	Non-Golfers Dominant Thigh *n* = 35	*p*-Value
Total thickness (cm)	4.45 ± 1.34	3.76 ± 0.82 *	0.017 *
	(2.60–7.80)	(2.12–5.81)	
Superficial non-contractile tissue layer (cm)	1.67 ± 0.79	1.58 ± 0.46	0.581
	(0.54–4.01)	(0.67–2.64)	
% Superficial non-contractile tissue	36.4 ± 9.4	41.8 ± 7.73	0.013 *
	(17.4–57.3)	(27.7–58.4)	
Muscle thickness layer (cm)	2.78 ± 0.73	2.18 ± 0.55	<0.0005 *
	(1.43–4.16)	(1.31–3.73)	
% Muscle thickness	63.6 ± 9.4	58.2 ± 7.73	0.013 *
	(42.7–82.6)	(41.6–72.3)	
Muscle thickness (cm) per kg	0.44 ± 0.13	0.30 ± 0.07	<0.0005 *
	(0.23–0.83)	(0.19–0.48)	

Values are mean ± SD (range). Superficial non-contractile layer comprised subcutaneous fat and superficial fascia; total muscle thickness with deep fascia between rectus femoris and vastus intermedius. *p*-values represent the difference between the golfers’ and non-golfers’ dominant thigh. * Difference between groups is statistically significant at the *p* < 0.05 level.

**Table 4:** Multiple regression analysis of ultrasound parameters, controlling for age and body mass index, in golfers and non-golfers.

Dependent Variable	B value	95% CI	*p* Value
Total anterior thickness	−0.984	−1.64 to −0.32	0.004 *
Non-contractile tissue	−0.364	−0.75 to 0.02	0.064
Non-contractile tissue %	1.987	−3.01 to 6.98	0.429
Muscle thickness	−0.619	−1.00 to 0.29	0.002 *
Muscle %	−1.987	−6.98 to 3.01	0.429

Abbreviations: CI, Confidence intervals; B value represents the difference between golfers and non-golfers, holding age and BMI constant. The independent variable is golfers compared to non-golfers. * Difference between groups is statistically significant at the *p* < 0.05 level.
